# Proton-pump inhibitor use is associated with a broad spectrum of neurological adverse events including impaired hearing, vision, and memory

**DOI:** 10.1038/s41598-019-53622-3

**Published:** 2019-11-21

**Authors:** Tigran Makunts, Sama Alpatty, Kelly C. Lee, Rabia S. Atayee, Ruben Abagyan

**Affiliations:** Skaggs School of Pharmacy and Pharmaceutical Sciences, University of California San Diego, La Jolla, CA USA

**Keywords:** Adverse effects, Alzheimer's disease, Peripheral neuropathies

## Abstract

Proton-pump inhibitors, PPIs, are considered effective therapy for stomach acid suppression due to their irreversible inhibition of the hydrogen/potassium pump in the gastric parietal cells. They are widely prescribed and are considered safe for over-the-counter use. Recent studies have shown an association between PPI use and Alzheimer dementia, while others have disputed that connection. We analyzed over ten million United States Food and Drug Administration Adverse Event Reporting System reports, including over forty thousand reports containing PPIs, and provided evidence of increased propensity for memory impairment among PPI reports when compared to histamine-2 receptor antagonist control group. Furthermore, we found significant associations of PPI use with a wide range of neurological adverse reactions including, migraine, several peripheral neuropathies, and visual and auditory neurosensory abnormalities.

## Introduction

Proton pump inhibitors (PPIs) are drugs commonly used in treatment of acid-related disorders including gastroesophageal reflux disease, *Helicobacter Pylori* induced gastric ulcers, duodenal ulcer, erosive esophagitis, and Zollinger-Ellison syndrome^1,2^. Treatment of acid-related disorders includes antacids, PPIs, and histamine-2 receptor antagonists (H2RAs)^3^. The PPIs are preferred over the H2RAs because of their superior efficacy due to their irreversible inhibition of the H+/K+ ATPase^4,5^. National Health and Nutrition Examination Survey (NHANES) revealed a rise in the number of PPI prescriptions (2.9–7.8%) among 40–64 year old individuals from 1999 to 2012^6^. NHANES did not account for over-the-counter (OTC) PPI intake. The class of PPI drugs includes six Food and Drug Administration (FDA) approved medications such as rabeprazole, lansoprazole, pantoprazole, esomeprazole, omeprazole, and dexlansoprazole. The high number of the PPI prescriptions, their OTC availability, and the increased likelihood of long-term use have raised concerns over unexpected adverse reactions (ADRs). It was demonstrated that the PPI pharmacology may not be limited to local inhibition of H-K-ATPase pump in parietal cells in the stomach^7,8^.

Common ADRs of PPIs, observed in clinical trials, include diarrhea, nausea, vomiting, flatulence, and headache^9–12^. Serious ADRs include breathing difficulty, rash, facial swelling, and throat tightness^9–12^. Recent studies revealed growing evidence of association with electrolyte abnormalities^13,14^ kidney injury^15,16^, bone fractures^17^, Clostridium difficile-associated diarrhea^18^, Alzheimer disease (AD)^19^, and non-AD type dementia^19,20^. However, other studies were not able to confirm the association between PPI use and a greater risk of dementia of both AD or non-AD type^21,22^.

Dementia associated with AD has a substantial impact on the quality of life of the patients and their caregivers^23,24^ and on the healthcare costs^25,26^. AD is considered the third most costly disease in the United States, with the costs being primarily associated with long-term care in nursing facilities^27^.

The current lack of consensus on PPI association with AD and non-AD type dementia warranted further investigation and analysis of other neurological outcomes. In our study, we performed an analysis of the FDA Adverse Event Reporting System database (FAERS/AERS) and identified significant increases of AD and non-AD dementia reports along with increased association with other types of memory impairment in PPI patients. Additionally, we found a significant increase in wide variety of peripheral neurological and neuropathic adverse events, as well as visual and auditory impairment ADRs.

## Results

### PPI “monotherapy” - neurological and neurosensory ADRs

**Figure 1:**
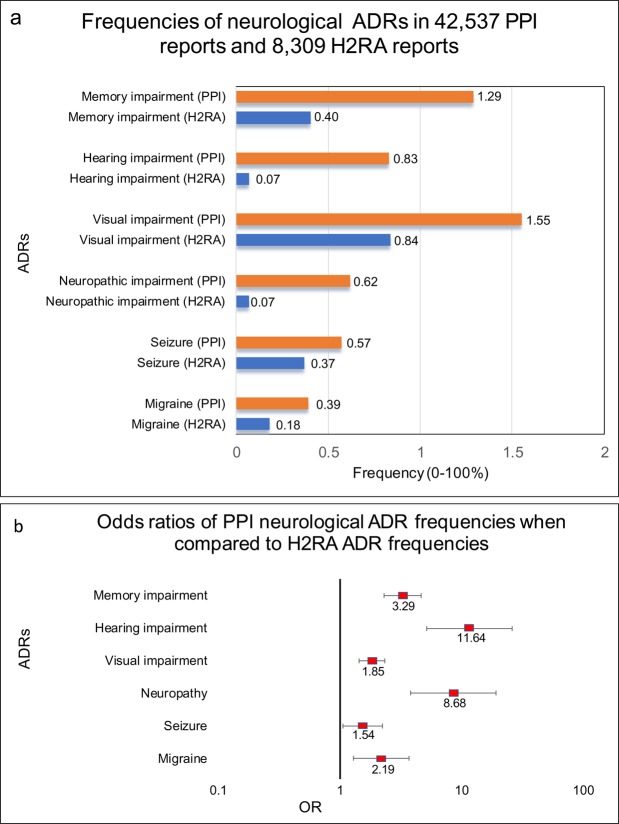
FAERS-reported frequencies and odds ratios of neurological/neurosensory adverse drug reactions. (**a**) Frequencies of neurological/neurosensory adverse events for patients in FAERS/AERS who took PPIs (n = 42,537) and H2RAs (n = 8,309). (**b**) Odds ratios were calculated comparing adverse event frequencies of PPI and H2RA patients. Ranges represent 95% confidence intervals (95% CI) (see Methods). A logarithmic X-axis shows odds ratios and their confidence intervals.

Reports in which PPIs were administered with no reported concurrent medications had a significant increase in memory impairment ADRs in comparison with H2RAs reports (OR 3.28, 95% CI [2.31, 4.67]) (Fig. 1b, Table 1). The outcomes included memory impairment, amnesia, dementia of the AD type, and non-AD dementia. Surprisingly, the H2RA cohort (n = 8,309) had three out of four ADRs listed above, but had no single report of dementia of the AD type (Table 1) while the PPI cohort (n = 42,537) had as many as 80 reports of the AD dementia. Interestingly, the auditory and visual ADRs followed a similar trend, with ORs being (11.64 [5.20, 26.11]) and (1.85 [1.44, 2.37]) respectively (Fig. 1b Tables 2 and 3). Neuropathic/neurological impairment ADR frequencies were also increased in the described above PPI cohort (8.68 [3.86, 19.49]) (Fig. 1b, Table 4). These included cranial and peripheral neuropathies, sciatica, and nerve injury as well as other neuropathic ADRs (Table 4). There was a small but significant increase in reported seizures (1.54 [1.06, 2.24]) (Fig. 1b and Table 5) and a significant increase in migraine reports in the PPI cohort (2.19 [1.29, 3.72]) (Fig. 1b and Table 6).

**Table 1:** Types and numbers of memory impairment (Memory impairment, amnesia, Alzheimer dementia, non-AD type dementia) related ADRs for patients on PPIs (n = 42,537) and H2RAs (n = 8,309).

Adverse drug reaction	PPI+	H2RA+
	(n = 42,537)	(n = 8,309)
memory impairment	246	11
amnesia	152	11
dementia, Alzheimer type	80	0
dementia, non-AD type	72	11
total memory impairment ADRs	550 out of 42,537	33 out of 8,309
odds ratio (95% CI) [p value]	3.29 (2.31 to 4.67) [p < 0.0001]	

Odds ratios were calculated from adverse event frequencies files.

**Table 2:** Types and numbers of hearing impairment related ADRs (hypoacusis, impaired hearing, deafness, unilateral deafness, sudden hearing loss etc.) for patients on PPIs (n = 42,537) and H2RAs (n = 8,309).

Adverse drug reaction	PPI+	H2RA+
	(n = 42,537)	(n = 8,309)
hypoacusis	134	2
impaired hearing	127	0
deafness	59	4
deafness unilateral	20	0
sudden hearing loss	12	0
deafness transitory	1	0
deafness neurosensory	1	0
deafness bilateral	1	0
total hearing impairment ADRs	355 out of 42,537	6 out of 8,309
odds ratio (95% CI) [p value]	11.64 (5.20 to 26.11) [p < 0.0001]	

Odds ratios were calculated from adverse event frequencies. ADRs reported as listed in the FAERS/AERS files.

**Table 3:** Reports containing ADRs related to visual impairment (visual impairment, blurred vision, blindness, reduced visual acuity, unilateral blindness etc.) for patients on PPIs (n = 42,537) and H2RAs (n = 8,309).

Adverse drug reaction	PPI+	H2RA+
	(n = 42,537)	(n = 8,309)
visual impairment	205	18
vision blurred	204	33
blindness	94	6
visual acuity reduced	82	5
blindness unilateral	34	1
visual field defect	18	1
visual disturbance	11	5
blindness transient	7	1
night blindness	2	0
sudden visual loss	1	0
total visual impairment ADRs	658 out of 42,537	70 out of 8,309
odds ratio (95% CI) [p value]	1.85 (1.44 to 2.37) [p < 0.0001]	

Odds ratios and 95% confidence intervals were calculated from adverse event frequencies and numbers of reports. The ADRs terms are taken directly from the FAERS/AERS files.

**Table 4:** ADRs related to neurological/neuropathic impairment (neuropathy, peripheral, nerve injury, nerve compression, sciatica, neuralgia, polyneuropathy, optic neuritis, hyperreflexia, peripheral sensory neuropathy etc.) for patients on PPIs (n = 42,537) and H2RAs (n = 8,309).

Adverse drug reaction	PPI+	H2RA+
	(n = 42,537)	(n = 8,309)
neuropathy peripheral	74	4
nerve injury	38	0
nerve compression	23	1
sciatica	21	0
neuralgia	14	0
polyneuropathy	12	0
optic neuritis	8	0
hyperreflexia	7	0
peripheral sensory neuropathy	5	0
IV-th nerve paralysis	5	0
VII-th nerve paralysis	5	0
autonomic neuropathy	4	0
peroneal nerve palsy	4	0
neurodegenerative disorder	3	0
areflexia	3	0
neurological symptom	3	0
optic ischaemic neuropathy	3	0
neuromyopathy	3	0
peripheral nerve injury	3	0
sciatic nerve injury	3	1
nerve degeneration	2	0
trigeminal neuralgia	2	0
cranial nerve disorder	2	0
neuritis	2	0
VI-th nerve paralysis	2	0
peripheral sensorimotor neuropathy	2	0
vagus nerve disorder	1	0
optic neuropathy	1	0
optic nerve injury	1	0
nerve root compression	1	0
nerve conduction studies abnormal	1	0
radial nerve palsy	1	0
neuropathy	1	0
neuropathic arthropathy	1	0
neurogenic bladder	1	0
motor neuron disease	1	0
hyporeflexia	1	0
CIDP	1	0
total neuropathic/neurological impairment ADRs	265 out of 42,537	6 out of 8,309
odds ratio (95% CI) [p value]	8.68 (3.86 to 19.49) [p < 0.0001]	

Abbreviations: CIDP - chronic inflammatory demyelinating polyradiculoneuropathy. Odds ratios and 95% confidence intervals were calculated from adverse event frequencies and numbers of reports. The ADRs terms are taken directly from the FAERS/AERS files.

**Table 5:** Types and numbers of seizure related ADRs (convulsion, seizure, epilepsy, grand mal convulsion, status epilepticus etc.) for patients on PPIs (n = 42,537) and H2RAs (n = 8,309).

Adverse drug reaction	PPI+	H2RA+
	(n = 42,537)	(n = 8,309)
convulsion	142	18
seizure	44	1
epilepsy	24	5
grand mal convulsion	10	2
status epilepticus	9	2
petit mal epilepsy	5	1
hypocalcaemic seizure	4	0
generalized tonic clonic seizure	2	0
partial seizures	1	2
hyperglycaemic seizure	1	0
juvenile myoclonic epilepsy	1	0
clonic convulsion	1	0
total seizure ADRs	244 out of 42,537	31 out of 8,309
odds ratio (95% CI) [p value]	1.54 (1.06 to 2.24) [p = 0.0237]	

Odds ratios were calculated from adverse event frequencies. ADRs reported as listed in FAERS/AERS files.

**Table 6:** ADRs related to migraine (migraine, migraine with aura, ophthalmoplegic migraine) for patients on PPIs (n = 42,537) and H2RAs (n = 8,309).

Adverse drug reaction	PPI+	H2RA+
	(n = 42,537)	(n = 8,309)
migraine	163	14
migraine with aura	4	1
ophthalmoplegic migraine	1	0
total migraine ADRs	168 out of 42,537	15 out of 8,309
Odds ratio (95% CI) [p value]	2.19 (1.29 to 3.72) [p = 0.0036]	

Odds ratios and 95% confidence intervals were calculated from adverse event frequencies and numbers of reports. The ADRs terms are taken directly from the FAERS/AERS files.

## Methods

### FDA adverse event reporting system

The FDA Adverse Event Reporting System (FAERS/AERS) was created by the FDA to document medication error reports, product quality complaints, and medication adverse events. Physicians, pharmacists, other healthcare providers, patients, and legal representatives submit the drug-related adverse event reports through MedWatch on a voluntary basis. If the adverse event is reported to the manufacturer, the manufacturer is required by law to forward the report to the FDA FAERS system.

At the time of data collection for the study, the FAERS/AERS contained over 10.3 million reports from January 2004 to March 2018. Both FAERS and AERS data sets are available online at: https://www.fda.gov/Drugs/GuidanceComplianceRegulatoryInformation/Surveillance/AdverseDrugEffects/ucm082193.htm.

All data analysis methods and procedures were carried out in accordance with existing guidelines and regulations. Since only the publicly available data were used in the study, and the FDA data sets used had been reviewed and released, no additional institutional and/or licensing committee approval was warranted.

### Combining and normalizing the FAERS/AERS reports

FAERS/AERS contains reports from the United States and other countries with their respective specific demographic formats and medication brand/generic names. Before the data collection and analysis online drug databases were utilized to create a dictionary with all of the varieties of drug brand names to translate them into generic names. FAERS/AERS quarterly report sets of seven files vary and their format had been changed at some point. To make the data sets more homogeneous, each quarterly report set of files was downloaded in dollar-separated text format (.txt) and modified to standardize the fields. Missing columns in the FAERS/AERS data set were added with no values to create a standard data table with over 10.3 million adverse event reports.

### Study outcomes

There were 20,317 uniquely worded ADRs reported to FAERS and AERS. ADRs were grouped into generalized categories of study outcomes: (1) *memory impairment* (memory impairment, amnesia, dementia Alzheimer type, dementia), (2) *neuropathy* (neuropathy peripheral, nerve injury, nerve compression, sciatica, neuralgia, polyneuropathy, optic neuritis, hyperreflexia, peripheral sensory neuropathy, IV-th nerve paralysis, VII-th nerve paralysis, autonomic neuropathy, peroneal nerve palsy, neurodegenerative disorder, areflexia, neurological symptom, optic ischaemic neuropathy, neuromyopathy, peripheral nerve injury, sciatic nerve injury, nerve degeneration, trigeminal neuralgia, cranial nerve disorder, neuritis, VI-th nerve paralysis, peripheral sensorimotor neuropathy, vagus nerve disorder, optic neuropathy, optic nerve injury, nerve root compression, nerve conduction studies abnormal, radial nerve palsy, neuropathy, neuropathic arthropathy, neurogenic bladder, motor neuron disease, hyporeflexia, and chronic inflammatory demyelinating polyradiculoneuropathy), (3) *visual impairment* (visual impairment, vision blurred, blindness, visual acuity reduced, blindness unilateral, visual field defect, visual disturbance, blindness transient, night blindness, and sudden visual loss), (4) *hearing impairment* (hypoacusis, impaired hearing, deafness, deafness unilateral, sudden hearing loss, deafness transitory, deafness neurosensory and deafness bilateral),(5) *migraine* (migraine, migraine with aura, and ophthalmoplegic migraine), and (6) *seizure* (convulsion, seizure, epilepsy, grand mal convulsion, status epilepticus, petit mal epilepsy, hypocalcemic seizure, generalized tonic-clonic seizure, partial seizures, hyperglycemic seizure, juvenile myoclonic epilepsy, and clonic convulsion).

### Cohort choice

**Figure 2:**
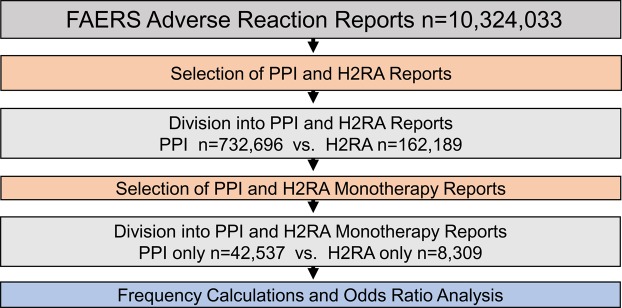
Legend: Flowchart of inclusion, exclusion and cohort selection for adverse event comparison between PPI and H2RA”monotherapy” reports.

For the PPI cohort (n = 732,696), out of 10,324,033 records, reports where rabeprazole, lansoprazole, pantoprazole, omeprazole and esomeprazole and dexlansoprazole were selected excluding reports with ranitidine, famotidine, cimetidine, and nizatidine concurrent use. For the H2RA cohort (n = 162,189), reports where ranitidine, famotidine, cimetidine, and nizatidine were used, excluding concurrent PPI use. The cohorts were further narrowed down to “monotherapy” reports. This assignment was defined when a submitted report contained a single PPI or a single H2RA medication. For that reason, any confounding concurrent medications and associated comorbidities were not applicable. Resulting cohorts consisted of 42,537 PPI reports and 8,309 H2RA reports (Fig. 2). ADR frequencies for both cohorts were calculated, and odds ratio analysis was performed by dividing their relative ADR frequencies and calculating the 95% confidence intervals of the OR values. The cohort choice was validated by strongly overlapping distributions of demographic parameters (Tables 7 and 8).

**Table 7:** Reported patient demographics in PPI and H2RA “monotherapy” cohorts.

Sex	PPI reports (n = 42,537)	Frequency (%)	H2RA reports (n = 8,309)	Frequency (%)	P-value	% Difference
Female	25116	59.69	4579	57.68	<0.001	2.01
Male	12000	28.52	2710	34.14	<0.001	5.62
Unreported	4963	11.79	650	8.19	<0.001	3.61
**Age difference**						
Mean age, years (SD)	58.3 (15.9)		55.6 (20.1)		<0.001	2.7
Median age	58.6		59.7		<0.001	1.1
Unreported (%)	45.4		55.1

**Table 8:** PPI and H2RA “monotherapy” report frequencies by country of origin.

Country	No. of PPI reports	Frequency %	No. of H2RA reports	Frequency %	% difference	>1% Different
United States	37139	88.26	6928	87.27	0.99	
Great Britain	1122	2.67	177	2.23	0.44	
Japan	472	1.12	382	4.81	3.69	*
Germany	357	0.85	24	0.3	0.55	
France	330	0.78	29	0.37	0.42	
Canada	309	0.73	22	0.28	0.46	
Italy	312	0.74	39	0.49	0.25	
Brazil	282	0.67	3	0.04	0.63	
Turkey	173	0.41	13	0.16	0.25	
Australia	151	0.36	7	0.09	0.27	
China	149	0.35	9	0.11	0.24	
Denmark	143	0.34	4	0.05	0.29	
Spain	138	0.33	13	0.16	0.16	
Nederlands	103	0.24	34	0.43	0.18	
Sweden	52	0.12	6	0.08	0.05	
Singapore	48	0.11	9	0.11	0	
Belgium	41	0.1	9	0.11	0.02	
New Zealand	42	0.1	3	0.04	0.06	
Chile	28	0.07	3	0.04	0.03	
India	15	0.04	15	0.19	0.15	
Costa Rica	30	0.07	0	0	0.07	
Unknown	198	0.47	45	0.57	0.1	

### Statistical analysis

#### Descriptive statistics

Frequencies for each studied side effect (Fig. 1a) was calculated by the equation:

1 $${\rm{Frequency}}=({\rm{nReports}}\,{\rm{with}}\,{\rm{ADR}}\,{\rm{in}}\,{\rm{a}}\,{\rm{cohort}})/({\rm{nReports}}\,{\rm{in}}\,{\rm{a}}\,{\rm{cohort}})\ast 100$$

#### Comparative Statistics

ADR report rates were compared via the Odds Ratio (OR) analysis for Fig. 1b and Tables 1–6 using the following equations:

2 $${\rm{OR}}=({\rm{a}}/{\rm{b}})/({\rm{c}}/{\rm{d}})$$

where

- Number of cases in exposed group with an adverse event.
- Number of cases in exposed group with no adverse event.
- Number of cases in control group with the adverse event.
- Number of cases in control group with no adverse event.

3 $${\rm{LnOR}}=\,\mathrm{Ln}({\rm{OR}})$$

Standard Error of Log Odds Ratio;

4 $${{\rm{SE}}}_{{\rm{LnOR}}}=\surd (1/{\rm{a}}+1/{\rm{b}}+1/{\rm{c}}+1/{\rm{d}})$$

95% Confidence Interval;

5 $$95 \% {\rm{CI}}=[\exp ({\rm{LnOR}}-1.96\times {{\rm{SE}}}_{{\rm{LnOR}}}),\exp ({\rm{LnOR}}+1.96\times {{\rm{SE}}}_{{\rm{LnOR}}})]$$

## Discussion

In our study, we analyzed a large number of ADR reports concerning patients taking only a single treatment of either a PPI and an H2RA drug using the FAERS/AERS databases, and quantified the association between PPI exposure and memory impairments, a wide range of neuropathies, visual and auditory impairments, migraines, and seizures.

### Memory impairment related ADRs

Using data from the German Study on Aging, Cognition and Dementia in Primary Care Patients 75 years and older, Haenisch *et al*. found that PPIs users were at increased risk of dementia (Hazard Ratio (HR) 1.38, 95% CI, [1.04–1.83]) and AD (HR, 1.44, 95% CI, [1.01, 2.06]) compared to non-users^19^. A later study lead by Gomm *et al*. analyzed data from the German statutory health insurer, Allgemeine Ortskrankenkassen (AOK) and observed a significantly increased risk of dementia (HR, 1.44, 95% CI, [1.36, 1.52], p < 0.001) in older adults using PPIs^20^. In contrast, Goldstein *et al*.^21^ analyzed data from Tertiary Academic Alzheimer Disease Centers and found that continuous and intermittent PPIs use was not associated with increased risk of dementia or AD^21^. In our study, we were not able to quantify the risk of AD-type dementia by itself since the H2RA cohort had zero reports of AD-type dementia and the PPI cohort had eighty reports. We included AD-type dementia in the generalized memory impairment cohort which included AD and non-AD type dementia, memory impairment and amnesia. We found a significant increase in memory impairment ADRs (OR 3.28, 95% CI [2.31, 4.67]) in the PPI cohort (Fig. 1b, Table 1). Memory impairment ADRs were helpful to capture possible early symptoms leading to dementia.

### Neuropathies

No large-scale clinical study has previously established a direct correlation of PPI use with neuropathies, including peripheral and poly-neuropathy, nerve injury and compression, sciatica, and neuralgia. Surprisingly, we identified a significant increase of neuropathy reports in patients taking only PPIs when compared to patients taking only H2RAs (OR 8.68, 95% CI [3.86, 19.49]). This observation was statistically significant, and clinically relevant. While our study did not address the molecular mechanism of action leading to neuropathies, a possible mechanism may involve Vitamin B12. An increased gastric pH level has been found to correlate with decreased Vitamin B12 levels^28–30^. In turn, B12 deficiency has been associated with reversible peripheral neuropathy and spinal cord degeneration and mental status alteration^31,32^. There are not many case reports depicting a direct correlation of PPI use with peripheral neuropathies^33,34^. While the direct correlation with PPI use and peripheral neuropathies had been noticed in a few case studies, we established an over eight-fold increase in broader range of neuropathy reports in a cohort of 42,537 PPI patients when compared to 8,309 H2RA patients.

### Hearing impairment

While a recent prospective study established an association between hearing loss with gastroesophageal reflux disease (GERD)^35^, no correlation with a specific antacid drug class was established. Furthermore, the study associated the hearing loss with the disease itself and suggested that PPI use may be beneficial for auditory ADRs. In other studies, the association between GERD and hearing problems was explained by the mid ear exposure to gastric acid in young patients with otitis media^36,37^. Contrary to these findings, our analysis showed a significant increase in risk of hearing impairment (OR 11.64 95% CI [5.20, 26.11]) specific to PPI-containing reports. This observation could not be explained by gastroesophageal disorders alone. Moreover, if this assumption were true, the hearing impairment risk would have been expected to be higher in the H2RA cohort since PPIs have superior efficacy in pH control. Thus, our finding supports a possible different mechanism in which the hearing impairment ADRs may be affected by the drug treatment and not the disease alone.

### Visual impairment

There have been many published case reports of visual impairment, including blurred vision^38^, ocular damage, optic neuropathy and blindness, associated with PPI use^39,40^. However, a retrospective cohort study of 94,063 patients, did not find an association between patients who took omeprazole *or* H2RAs (cimetidine, famotidine, nizatidine, ranitidine) and visual impairment^41^. In our analysis we divided the treated patients into two groups, compared their ADRs (42,537 PPI reports vs. 8,309 H2RA reports) and found an increased risk of visual impairment in patients taking PPIs (OR 1.85, 95% CI [1.44, 2.37]). This large-scale data analysis supports the initial case studies and provides a possible explanation the negative results of García Rodríguez *et al*. study in which PPI- and H2RA-treated patients were in a mixed cohort.

### Migraine

Associations between PPI use and headache were established in the clinical trials of all the approved PPIs^9^, but there were no reports specific to migraine which is associated with a higher burden on quality of life^42,43^.

The headache association was confirmed in a crossover study conducted in Taiwan where lansoprazole and esomeprazole use increased headache incidence (OR, 1.20, 95% CI, [1.07,1.35], P < 0.002)^44^. However, according to our findings the headache effect may be common to both treatment classes, since there was no significant difference in headache reports between PPI and H2RA cohorts. Surprisingly the migraine report frequencies were different between the cohorts. We observed a significant increase in migraine reports in the PPI monotherapy cohort (OR 2.19, 95% CI [1.29, 3.72]). To our knowledge this is the first study showing association between PPI use and risk of migraine.

### Seizures, convulsions, and epilepsy

An earlier observational cohort study found no association between PPI use and seizure risk in the overall population or in patients with epilepsy. In contrast, the study did find an increased risk of seizures in H2RA users^45^. On the other hand, in a few case reports the PPIs were found to be associated with seizures, and the authors attributed the seizure to PPI induced hypomagnesemia and hypocalcemia (electrolyte abnormalities known to influence neuronal function)^46–48^. Our analysis confirmed a small but significant association of PPI use with seizure-related ADRs (OR 1.54 95% CI [1.06, 2.24]) (Fig. 1b and Table 5).

### Practical implications

The observed risk of memory impairment, neuropathy, hearing and visual impairment, seizures as well as migraines with PPIs warrant a more careful consideration when selecting this class of medications for patients who already have or may be at high risk for these adverse effects, in particular, if the treatment exceeds the recommended duration limit of two or three weeks. Given these ADR observations and the availability of alternatives to PPIs for related conditions, the risk versus benefit should be carefully considered before initiating a PPI. When clinically indicated, PPIs should only be used for the duration recommended by FDA and not exceed it. The latter may be difficult to enforce because of the OTC availability of some PPIs. Additionally, other medications that may exacerbate these ADRs should be avoided when patients are prescribed PPIs. Long-term use of PPIs beyond eight weeks can be considered in individual cases as long as the benefits continue to outweigh the risks. During PPI treatment, patients should be educated about potential ADRs and advised to contact the prescriber if they suspect any of the ADRs listed above.

## Conclusion

This is the first large-scale postmarketing study to show significant association between PPI monotherapy and neurological and neurosensory ADRs. Further prospective clinical trials should evaluate the neurological and sensory ADRs. In the meantime, caution and awareness of these potential ADRs are recommended. H2RAs and other treatment modalities may be considered in patients at high risk for developing memory impairment, neuropathy, hearing and visual impairment, or migraines.

### Study limitations

Since FAERS and AERS reporting is voluntary, the data set represents only a subset of actual cases and therefore the FAERS/AERS ADR frequencies should not be confused with absolute population incidences. FAERS/AERS reporting can be biased by newsworthiness, and scientific and legal variables^49,50^. Recent studies have shown that some adverse events in the FDA database are significantly underreported^49,51^. An acceptable way to deal with this issue was to use Odds Ratios with 95% CI between two cohorts instead of relying on ADR frequencies in a single cohort. Other limitations stem from the occasionally missing demographic variables, treatment dose and duration, and lack of comprehensive medical record data.

Although the study design was modeled to exclude potential confounding factors by selecting monotherapy reports, some concurrent medications and comorbidities may be also underreported, which in turn may affect the PPI and H2RA cohort composition and statistical analysis. Despite the fact that in clinical and community practice medicine reconciliation is becoming increasingly implemented, accounting for over-the-counter medication and supplement use still remains a significant unknown variable as it relies on patient self-reporting. This variable is a limitation at all the levels of clinical research from case studies to most controlled clinical trials. This is an association study where the physiological mechanism of the ADRs cannot be derived from the records. The causality of the ADRs cannot be inferred from association alone.
